# Positioning Performance of BDS Observation of the Crustal Movement Observation Network of China and Its Potential Application on Crustal Deformation

**DOI:** 10.3390/s18103353

**Published:** 2018-10-08

**Authors:** Xiaoning Su, Guojie Meng, Haili Sun, Weiwei Wu

**Affiliations:** 1 Key Laboratory of Earthquake Prediction, Institute of Earthquake Forecasting, China Earthquake Administration, Beijing 100036, China; suxiaoning_666@126.com (X.S.); 14_jasonwu@tongji.edu.cn (W.W.); 2 College of Surveying and Geo-Informatics, Tongji University, Shanghai 200092, China; 3 College of Resourse Environment and Tourism, Capital Normal University, Beijing 100048, China; sunhaili@cnu.edu.cn

**Keywords:** BDS, CMONOC, data quality, positioning accuracy, BDS derived velocity field

## Abstract

The Crustal Movement Observation Network of China (CMONOC) has begun receiving BeiDou Navigation Satellite System (BDS) observations since 2015, and accumulated more than 2.5 years of data. BDS observations has been widely applied in many fields, and long-term continuous data provide a new strategy for the study of crustal deformation in China. This paper focuses on the evaluation of BDS positioning performance and its potential application on crustal deformation in CMONOC. According to the comparative analysis on multipath delay (MPD) and signal to noise ratio (SNR) between BDS and GPS data, the data quality of BDS is at the same level with GPS measurements in COMONC. The spatial distribution of BDS positioning accuracy evaluated as the root mean square (RMS) of daily residual position time series on horizontal component is latitude-dependent, declining with the increasing of station latitude, while the vertical one is randomly distributed in China. The mean RMS of BDS position residual time series is 7 mm and 22 mm on horizontal and vertical components, respectively, and annual periodicity in position time series can be identified by BDS data. In view of the accuracy of BDS positioning, there are no systematic differences between GPS and BDS results. Based on time series analysis with data volume being 2.5 years, the noise characteristics of BDS daily position time series is time-correlated and corresponding noise is white plus flicker noise model, and the derived mean RMS of the BDS velocities is 1.2, 1.5, and 4.1 mm/year on north, east, and up components, respectively. The imperfect performance of BDS positioning relative to GPS is likely attributed to the relatively low accuracy of BDS ephemeris, and the sparse amount of MEO satellites distribution in the BDS constellation. It is expectable to study crustal deformation in CMONOC by BDS with the gradual maturity of its constellation and the accumulation of observations.

## 1. Introduction

As an important participator of the Global Navigation Satellite System (GNSS), BeiDou Navigation Satellite System (BDS) is designed to provide positioning, navigating and time services in the world. BDS is formed by three types of satellites including five Geostationary Earth Orbit (GEO) satellites, three Inclined Geosynchronous Satellite Orbit (IGSO) satellites, and 27 Medium Earth Orbit (MEO) satellites. By the end of 2012, there were five GEO satellites, five IGSO satellites and four MEO satellites in the integrated constellation of BDS, making it capable to provide services in Asia-Pacific area. Since the end of 2012, an additional one GEO satellite and 14 MEO satellites have been launched in order to fill in the incomplete BDS constellation. A fully integrated BDS constellation, consisting of 35 satellites, is to be fulfilled in 2020 and to make global services come true (http://www.beidou.gov.cn). Previous studies have widely studied the capacity, the quality and the accuracy of BDS, with their evaluation of the accuracy of BDS code and phase measurements being 33 cm and 2 mm, respectively, which are at the same level with GPS measurements [1,2,3,4,5]. BDS satellite has been widely applied in many fields [6]. BDS-Reflectometry was used to estimate sea level changes [7]. GEO satellites have large potential for continuous monitoring of space weather effects in low-latitude and equatorial ionosphere [8]. Till now, the positioning, navigating and time services of BDS have already reached or even exceeded its designed targets [9,10,11,12,13,14]. 

The Crustal Movement Observation Network of China (CMONOC) consists of 260 continuous stations and 2000 campaign stations, which can provide elaborate three-dimensional velocity field and serve as important tools to study the characteristic of crustal deformation and strain accumulation in China mainland and surrounding areas [15,16]. Since 2015, some CMONOC continuous stations have started to receive BDS signals in order to implement the application of BDS services. Reference [14] compared the positioning results between GPS and BDS, and made the conclusion that the geodetic reference frame established and maintained by BDS can reach the need of centimeter-level accuracy, and proposed the urgent needs on deploying and updating more GPS/BDS dual module receivers in CMONOC. It is necessary to evaluate the quality of the BDS observations and its positioning performance in CMONOC [17,18].

This paper aims to evaluate the positioning performance of BDS with observations collected in CMONOC. In Section 2, we detail BDS data processing for daily solution. In Section 3, we show the quality of BDS measurements seriatim, evaluate the accuracy of BDS position time series, make comparisons with GPS results, depict the spatial distribution of differences between BDS and GPS positioning results, and derive three-dimensional BDS velocity field. In Section 4, we try to find the reason causing the relatively low performance of BDS positioning. In Section 5, we make a conclusion of this paper.

## 2. Materials and Methods 

### 2.1. BDS and GPS Data

**Figure 1:**
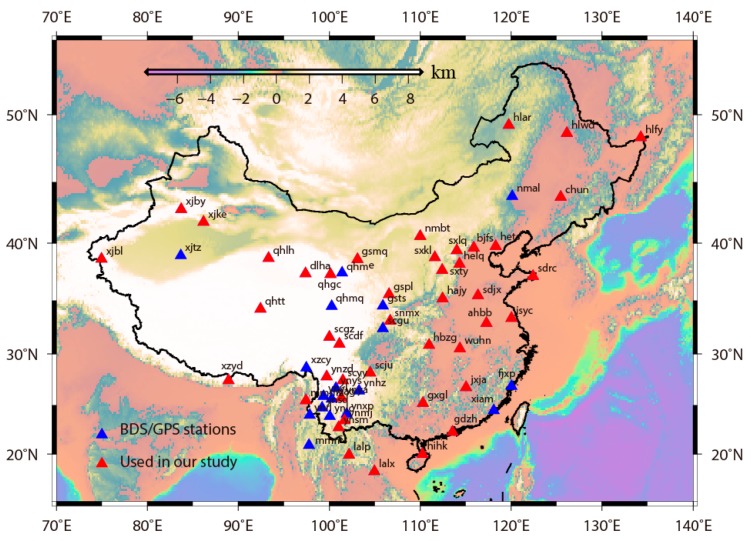
The distribution map of the station deployed with BDS/GPS dual mode receivers. The blue and red solid triangles represent the location of BDS/GPS dual-frequency stations, with the total number being 62. The red solid triangles are stations analyzed in this study, with the number being 44 and their data integrity being over 95% in the period of the analysis.

The CMONOC consists of 260 continuous stations, 28 of which were installed in 1999, the remaining sets of which have operated since 2010, and 62 of which have deployed with BDS/GPS dual module Trimble NETR9 receivers since 2015. The raw data can be obtained by asking the data center of CMONOC (http://www.neis.cn/). Figure 1 shows the spatial distribution of 62 BDS/GPS dual module stations. A total of 884 daily results are estimated with BDS and GPS observations from day 213 of 2015 to day 365 of 2017. The first 60 solutions in 2017 and 44 stations with almost full-time occupation history were used to estimate the accuracy of positioning results from different satellite systems. The signals from 14 BDS satellites can be tracked. However, due to the extraordinary large clock errors in C02 satellite of GEO, 13 BDS satellites, including 4 GEO, 5 IGSO, and 4 MEO satellites, were included during the data processing in this study. 

### 2.2. Data Processing Strategy

There are two classical modes, including the relative positioning and precise point positioning, in the GNSS data processing domain. The relative positioning can eliminate all satellite ends and station ends errors, and improve the resolution of phase ambiguities. The relative positioning algorithm in GAMIT/GLOBK package version 10.6, with BDS data handling function enabled, is employed in this study [19,20]. The same setting of parameters and strategies of data processing was applied for both BDS and GPS data in order to avoid strategically errors in the comparison of positioning results. The ionosphere-free combination was introduced in forming observation equations to eliminate the first-order ionosphere delay effects, and the second-order ionosphere effects are corrected by models reported by Petrie et al. [21]. As the second-order ionosphere effects are correlated with the total electron content and geomagnetic field, the Vertical Total Electron Content (VTEC) maps from the Center for Orbit Determination in Europe (CODE) and the newly updated geomagnetic field model IGRF12 from the international geomagnetic reference, were applied to eliminate their influences [21]. The VMF1 mapping function was employed in the modeling of the dry part of troposphere delay effects, with atmosphere pressure and temperature parameters collected from station-wise meteorological files and GPT2 grid files, to improve the accuracy of estimated station position parameters on the vertical component [22]. The wet part of troposphere delay effects was set as unknown hourly parameters in the final estimation of position coordinates, together with two daily gradient parameters in the north-south and east-west directions, respectively. The IGS14 absolute phase center model was applied to correcting the variation of phase center for satellites and receivers. Satellite final orbits were determined with ephemeris produced by Wuhan University (ftp://ics.gnsslab.cn), which include BDS and GPS satellite ephemeris. Other classical parameters and models are all referred to the newly updated results from MIT (http://www-gpsg.mit.edu). The sampling frequency was set to 30 s, and the cut off of elevation angle is 10°. During the data processing stage, station position and earth rotation parameters were simultaneously estimated as unknowns in the final loosely constrained daily solution. 

**Figure 2:**
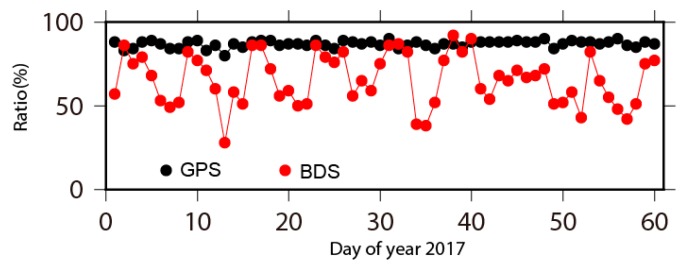
The ratio of ambiguity resolution from both GPS and BeiDou Navigation Satellite System (BDS) data.

Figure 2 shows the ratio of ambiguity determined as integer value for both GPS and BDS observations, respectively. A total of 86.9% daily ambiguities are fixed to be integers for GPS observations. The number drops to 66.8% for BDS observations on average, together with significant temporal variations. The scope of BDS ambiguity resolution is about 40–90%, which may be attributed to the temporally uneven distribution of BDS satellites [23].

Two sets of quasi-observation files (H files) are produced on GAMIT for BDS and GPS observations, respectively. To finally resolve station position parameters, we used the daily constrained bias-fixed solutions of the parameters and their associated covariance matrices as quasi-observations in GLOBK to generate position time series in the International Terrestrial Reference Frame 2014 (ITRF2014). Three types of combination strategies, GPS, BDS and GPS/BDS combined were implemented. In the GPS/BDS combined solution, the weights of both H files were set to be equal due to their similar quality of observations. Based on the minimum constraints principle, the reference frame of station position coordinates was aligned to ITRF2014 through the 6-parameters similarity transformation of 8 evenly distributed stations in CMONOC (BJFS, XJBL, QHLH, YNZD, WUHN, JXJA, NMBT, and HLAR) [24]. We used four iterations to eliminate bad stations and to compute station weights for the reference frame stabilization.

The derived position time series were modeled to estimate a constant velocity term together with annual and semiannual sinusoidal variation for each component reported by reference [25]. A total of 29 stations position time series, with available data percentage exceeding 90% in 884 days, were modeled by secular trends and seasonal signals for both BDS and GPS results. Outliers are iteratively deleted by using the interquartile range (IQR) algorithm described by reference [25]. Noise model of time series can efficiently affect the estimation of velocity field, especially on the accuracy of estimated trends. Color noise has been confirmed to be contained in the GPS position time series [26,27,28]. We employ HECTOR software to estimate the power index of the noise model for 29 continuous station position time series [29]. The results of the noise analysis show that the corresponding noise model is white plus flicker noise model. Thus, to precisely estimate the velocity field from both BDS and GPS station position time series, we applied white plus flicker noise model to forming the covariance matrix for the least squares adjustment of secular trends.

## 3. Results

The accuracy of BDS positioning results was evaluated as the RMS of station residual position time series, with secular trends and non-tectonic signals removed from the original station position time series. Due to larger data volumes, continuous observations collected since 2010, and data accumulation more than 5 years, GPS data was used to simulate secular trends and non-tectonic signals. We used classic function model from which contains one constant, one secular, two seasonal and two semi-seasonal parameters, to fit GPS station position time series, and deduct corresponding model values from original BDS and GPS position time series to acquire the target residual time series [25]. We used the least square algorithm to estimate parameters. 

### 3.1. Quality Analysis of BDS Observations

**Figure 3:**
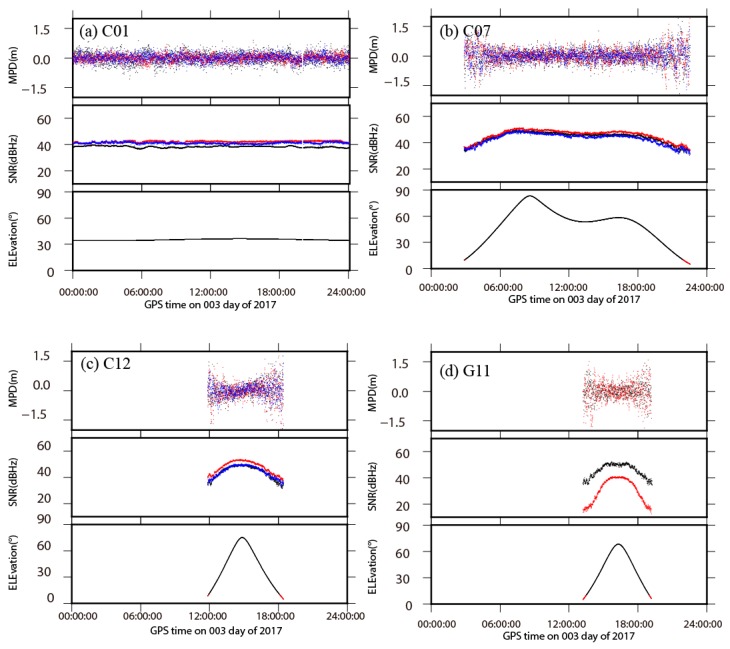
The multipath delay (MPD), signal to noise ratio (SNR) of BDS and GPS satellite systems at station YNSM, and the variation of their elevation angles with respect to time. (**a**) C01 (GEO), (**b**) C07 (IGSO), (**c**) C13 (MEO) and (**d**) G11 (GPS) are selected as typical examples on behalf of different satellite types. Different colors represent different frequencies. Red circle, black circle, and blue circle represent signal B1, B2, and B3, respectively, in (**a**–**c**). Red circles and Black circles represent the signal L1 and L2, respectively, in (**d**).

The station YNSM is selected as the example to specify the status of observing and quality of observation for BDS data in CMONOC. The number of tracking satellites on one single epoch is 9–13 for BDS and 8–12 for GPS, respectively (Figure S1). In order to evaluate the quality of BDS data, the MPD and SNR of three sets of BDS satellites and GPS satellites are shown in Figure 3. The multipath delay includes two parts: The delay of satellite ends and the delay of ground station ends. The MPD in satellite ends is caused by the spacecraft internal multipath, and the impedance mismatch between antenna elements and power divider network causes reflection signals. Piecewise linear [30] or third-order polynomial [31] models can effectively mitigate or even eliminate the effects of MPD in satellite ends [32,33,34]. The magnitude of the MPD in station ends is related to the distance between the antenna and the object, the angle of the reflected signal, and the characteristics of the reflector. We used the MPD in station ends associated with observation condition to analyze the data quality in CMONOC. The SNR and MPD are highly correlated with the elevation angles of satellites, and the magnitude of MPD decreases with the increase of satellite elevation angle. The MPD for different frequencies are similar with each other, while the SNR are significantly distinct, especially for the GPS satellites, with larger SNR on L2 data comparing to L1 data. 

The variation of MPD and SNR for B1 data and L1 data from four different satellites with respect to satellite elevation angle are shown in Figures S2 and S3. The MPD of B1 and L1 data decline with the increase of satellite elevation angle, and their quantities are at the same level. The specific RMS for C01 (GEO), C07 (IGSO), C12 (MEO) and G11 (GPS) are 0.26 m, 0.38 m, 0.46 m, and 0.47 m, respectively, and the last two values confirm the similarity on the status of observing and the quality of observations between GPS and MEO. The elevation angles of GEO satellites are always near 35°, while they vary widely for IGSO and MEO satellites. The large RMS of MPD for MEO and IGSO, comparing with GEO, are attributed to their low elevation angles. Due to longer periods with the observing condition of low elevation angles for MEO, the corresponding RMS is larger than IGSO. Therefore, we can largely conclude that the RMS of MPD is directly correlated to the elevation angles of satellites in daily sessions. The SNR for IGSO, MEO, and GPS are positively correlated to the satellite elevation angle in similar correlation patterns. GEO satellites have smaller SNR comparing with IGSO, MEO and GPS on the same elevation angle, and possess almost stationary quantities due to their fixed elevation angles. 

### 3.2. The RMS of Station Residual Position Time Series

**Figure 4:**
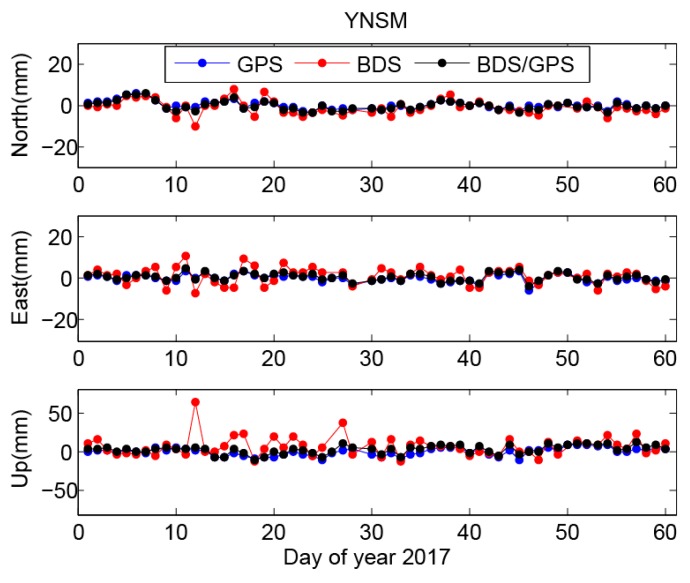
The residual position time series at station YNSM for BDS, GPS, and GPS/BDS combined solutions, respectively.

Figure 4 shows the residual position time series of station YNSM, which are derived from BDS, GPS, and BDS/GPS data, respectively. Three residual time series show similar steady temporal behaviors, especially for the GPS and BDS/GPS results, which are nearly complete coincidence. The BDS residual time series has the large temporal dispersion and contains several abnormal jumps, which is not found in GPS or BDS/GPS results.

The RMS of GPS residual position time series for station YNSM is 2.5, 2.1, and 5.1 mm on north, east, and up components, respectively. The corresponding RMS of BDS/GPS result is 2.1, 1.9, and 4.3 mm, respectively, which indicates better inner accuracy of station positions from the combined estimation for BDS and GPS results. The RMS of BDS result is 3.4, 3.9, and 12.6 mm, respectively, which are significantly lower than GPS or BDS/GPS results. We present the average RMS of 44 CMONOC stations in Table 1. Comparing to the GPS results, the average RMS of BDS/GPS residual position time series shows a reduction of 20% and 13.5% for horizontal and vertical components, respectively. The average RMS is 6.1, 7.1, and 22.1 mm for BDS results on north, east, and up components, respectively. The result indicates the accuracy of BDS positioning is holistically lower than that of GPS and BDS/GPS results.

### 3.3. Differences Between BDS and GPS Position Time Series

In order to recognize the systematic discrepancies between BDS and GPS positioning results, we present the mean and RMS of differences between BDS and GPS position time series. As an example, the mean of differences between BDS and GPS results for station YNSM is −0.6, −1.0, and −3.8 mm for north, east, and up components, respectively. The corresponding RMS is 7.1, 8.5, and 19.4 mm, respectively. We present the summary of the mean and RMS from 44 position differential time series between BDS and GPS results in Table 2. The mean values are −0.8, −1.5, and −5.5 mm for the north, east, and up components, respectively, and the corresponding RMS are 6.8, 7.3, and 21.7 mm, respectively. We estimated the seven parameters of coordinate transformation between BDS and GPS daily position time series by least square sense, and obtained daily seven transformation parameters. The magnitude of the mean of translation and rotation parameters is almost near zero, while the mean scale is 2.2 ppb (ppb equals 10^−9^), indicating the root cause of discrepancy between BDS and GPS results is the change of scale. The average RMS of BDS residual position time series, which is about 7 mm and 22 mm on horizontal and vertical components, respectively, are at the same level with the RMS of differential position time series. There are no systematic differences between BDS and GPS position time series. 

### 3.4. The Spatial Distribution of Differences Between BDS and GPS Positioning Results

We present the position residual time series for station YNSM (south China), station AHBB (east China), station QHGC (north China) and station XJBY (west China) from three sets of positioning results in Figure 4 and Figure S4, respectively. There are always several abnormal jumps in BDS residual time series for stations in every territory of China, which is unseen for GPS results. The maximum horizontal difference can reach 30 mm, and the maximum vertical difference can reach 80 mm. The BDS/GPS station position time series are coincidence with GPS results everywhere. As shown in Figure S1, 13 BDS satellites are used for positioning, four GEO of them are almost fixed in space, and only include four MEO satellites which is a similar trajectory pattern and positioning contribution with that of GPS satellites. However, 32 GPS satellites are involved in positioning, and the spatial geometric distribution of GPS satellites is more reasonable than that of BDS satellites. Therefore, we think BDS observation is a limited supplement for combined BDS/GPS positioning under the current BDS MEO satellite distribution, and this status maybe results in the similar results between GPS and BDS/GPS observations.

**Figure 5:**
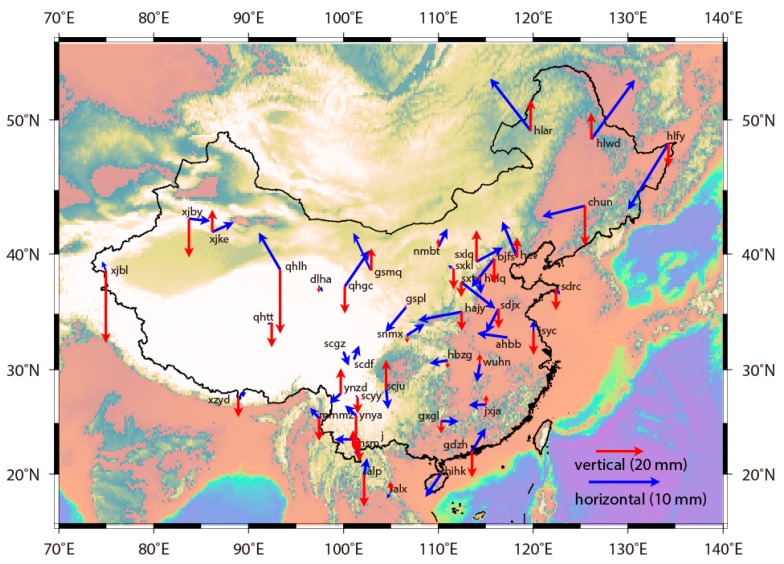
The spatial distribution of mean values on differences between BDS and GPS positioning results.

To elaborately specify the characteristic of the spatial distribution for differential time series between BDS and GPS results, we present the mean value of differential time series from 44 CMONOC stations on horizontal and up components, respectively, in Figure 5.

We try to investigate the spatial pattern of the direction of the mean horizontal differences. The directions of mean horizontal differences for station XJBY and station XJKE are both eastward, while they are northwestward for station XJBL, which is on their southwestern area, and station QHLH, which is on their southeastern area. The mean horizontal differences are nearly nil for station DLHA, station XZYD and station QHTT, while significant value points to northeast direction for station QHGC, which is not far away from station DLHA. These two examples indicate that it is random without any disciplinary pattern for the spatial distribution of the direction of mean horizontal differences. In southwest China, south China, north China and northeast China areas, the directions are all randomly distributed, thus confirming the aforementioned conclusion.

We intend to identify the spatial pattern of the quantity of the mean horizontal differences. It is ~3 mm for the quantity of the mean horizontal differences in the district southern of N30°, ~3–8 mm in the district between N30° and N43°, and ~10–15 mm in the district northern of N43°. The different quantities of the mean horizontal differences in China indicate their enlargement pattern and the decline in the accuracy of BDS positioning results with the increasing on the latitude of the station. Reference [35] concluded that the accuracy of GEO positioning shows a declining pattern with the increasing latitude of the station, which is origin from the fixation of five GEO satellites in the southern sky-area of China. Analogously, the visibilities of five IGSO satellites have similar declining patterns with the increasing latitude of the station. Thus, due to the non-uniform distribution of GEO and IGSO satellites, the accuracy of BDS positioning results shows significant spatial latitude-dependent feature, having a declining pattern with the increasing latitude of the station [36].

We analyzed the spatial pattern of the mean vertical differences in CMONOC. The largest mean vertical difference, with its quantity being 30 mm, comes from the westernmost station XJBL, and they show opposite directions at nearby station XJBY and station XJKE. In other districts, they also show random patterns. Stations with infinitesimal value of the mean vertical differences are spatially randomly distributed: Station DLHA on the northeast margin of Tibetan plateau, station NMBT on the Mongolian plateau, station SNMX and station HBZG in the south China block. As towards the absolute quantities of the mean vertical differences, they are ~8–12 mm in southwest China district, ~10–12 mm in north China district, ~10–15 mm in northeast China district, indicating no spatial latitude-dependent pattern, ~8–30 mm in Xinjiang district, ~10 mm in Gansu district, indicating also no spatial longitude-dependent pattern.

### 3.5. The Three-Dimensional Velocity and Its Accuracy Deduced by BDS and GPS Observations, Respectively

The accuracy of estimated velocity fields for both BDS and GPS position time series is displayed in Table 3. The average accuracy of the BDS velocity field are 1.2, 1.5, and 4.1 mm/year on the north, east and up components, respectively, while they are 0.3, 0.3, and 0.7 mm/year for the GPS velocity field, which are remarkably smaller, indicating high accuracy of the GPS velocity field.

**Figure 6:**
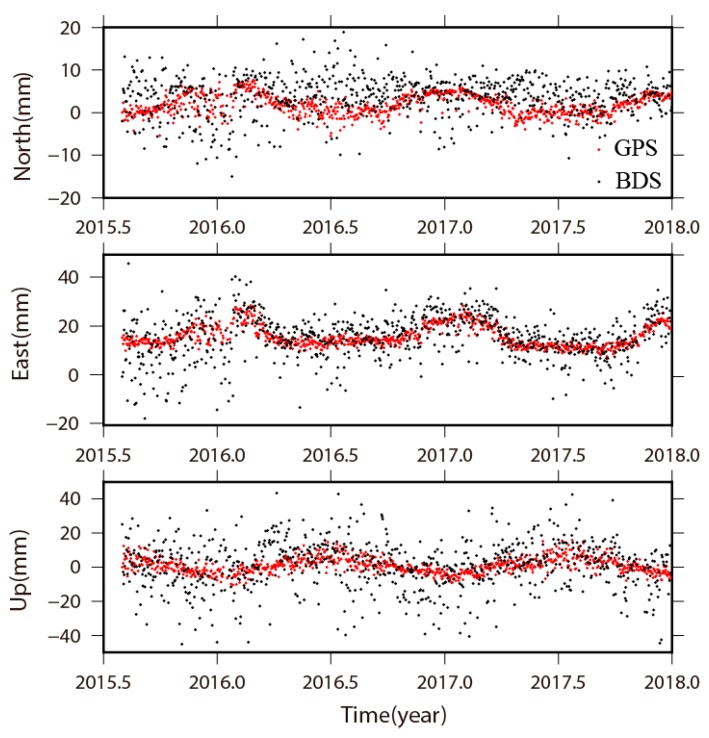
The trend free position time series of BDS and GPS results at station SXTY located in Shanxi Province of China.

In Figure 6, we present the trend free time series at station SXTY for both BDS and GPS results, which is located in Shanxi Province of China. There are notable seasonal signals that emerged in the trend free time series at Station SXTY. The dispersion of the BDS result is larger than GPS. For the GPS result, the estimated annual amplitudes are 5.1, 2.4, and 4.5 mm on the north, east and up components, respectively. For the BDS results, the corresponding annual amplitudes are 6.1, 0.5, and 6.2 mm. As the annual amplitudes are remarkable on the north and vertical components, the annual signals are visible in both BDS and GPS position time series with similar amplitudes estimated. While it is unremarkable on the east component, estimable in GPS results and invisible in BDS results, thus resulting in different estimation of amplitudes. To summarize the feature of seasonal signals at all 29 stations, for GPS results, 15 stations have significant annual variations on the horizontal components for GPS results, and 13 stations possess clear annual variations on the vertical component for GPS results. These annual signals can also be visible and well estimated for BDS results.

**Figure 7:**
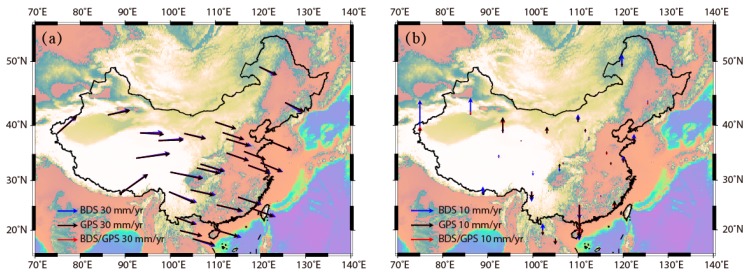
The velocity field derived from BDS/GPS stations derived from GPS, BDS and BDS+GPS data in International Terrestrial Reference Frame 2014 (ITRF2014). (**a**) Horizontal component. (**b**) Vertical component.

The velocity fields aligned to the ITRF2014 reference frame for BDS, GPS and BDS/GPS combined results, are presented in Figure 7. It is negligible for the differences between GPS results and BDS/GPS combined results. However, they are remarkable for the differences between GPS velocity field and BDS velocity field, with maximum horizontal differences reaching up to 1.6 mm/year, which is similar with the estimated accuracy of BDS velocity field. The values of vertical differences are less than the vertical accuracy of BDS velocity field at 27 stations, and the direction of vertical differences are also randomly distributed, thus indicating that it is insufficient to derive precise vertical velocity fields with BDS results in CMONOC.

## 4. Discussion

### 4.1. Influence of Satellite Orbit Accuracy on Positioning Performance

Satellite orbit parameters are fixed with final precise ephemeris products in this study, which are the most strongly recommended processing strategy in processing of regional or local GNSS network [19]. Reference [37] showed that the radial accuracy of orbits for BDS satellites can reach to 10 cm. Reference [38] also concluded that the overlapped RMS on the radial component of the orbits for BDS satellites are 10 cm. Reference [39] proved that the 3 days-session overlapped radial RMS can reach up to 1.8 m and 0.3 m for GEO and IGSO/MEO, respectively, however, the radial accuracies are 10 cm for all kinds of BDS satellites.

**Figure 8:**
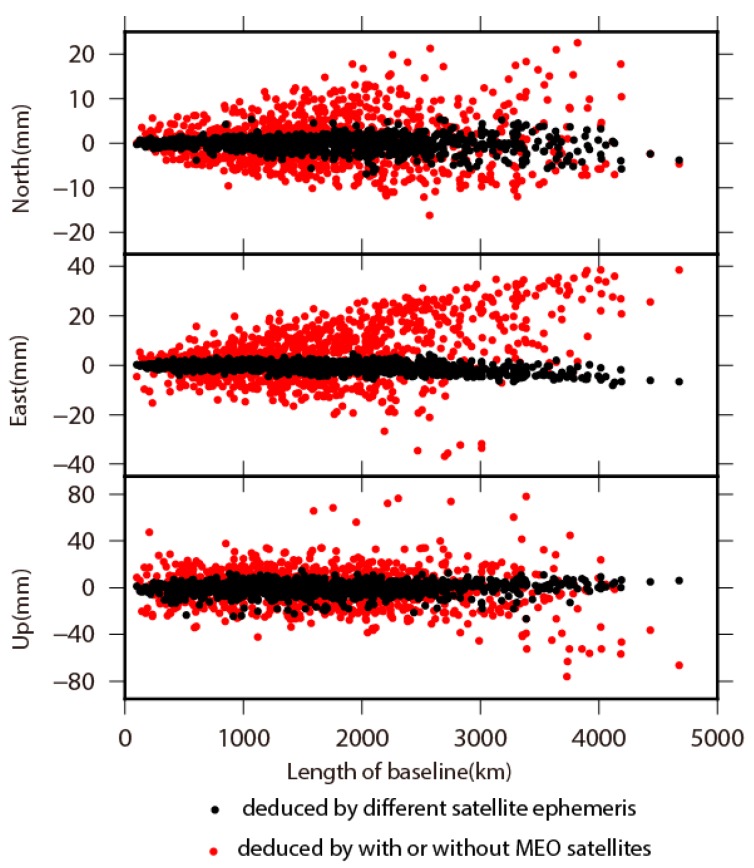
The variations on average values of baseline differences caused by different satellite ephemeris and whether or not including MEO satellites in BDS processing, with respect to baseline length.

**Figure 9:**
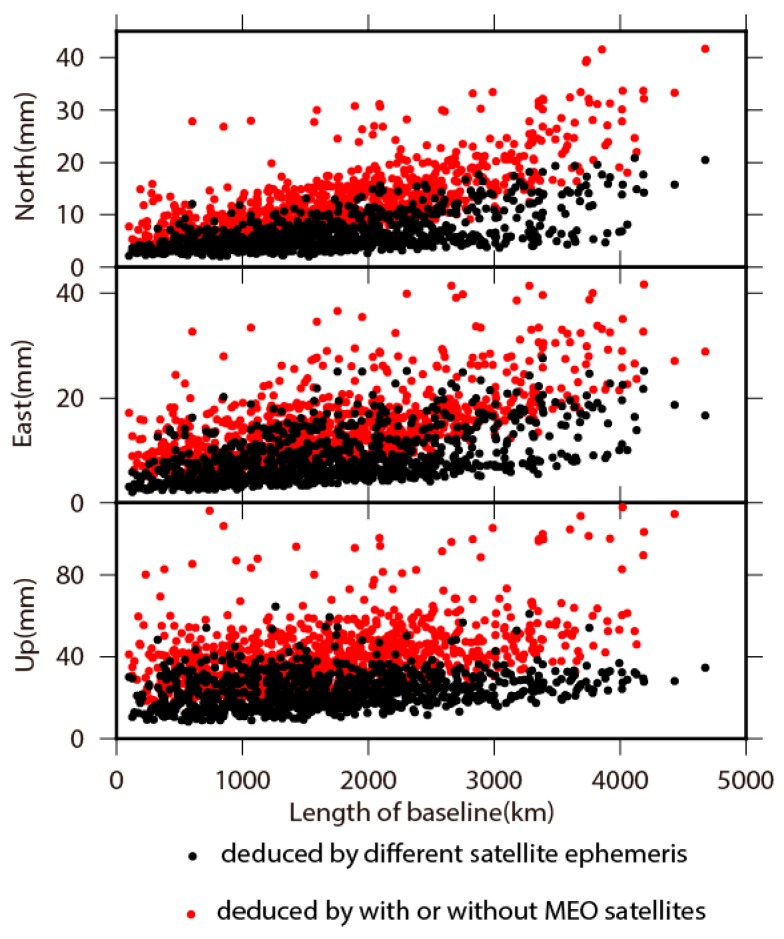
The variations on RMS of baseline differences caused by different satellite ephemeris and whether or not including MEO satellites in BDS processing, with respect to baseline length.

To clarify the influence of ephemeris for BDS satellites, we acquired two sets of baseline solutions on GAMIT based on Wuhan University and GFZ ephemeris respectively, and made comparisons between them. In Figure 8 and Figure 9, we present the mean and RMS of the differences between these two sets of baseline solutions. The mean of the differences has a scope of from −6.9 to 5.4, from −8.1 to 4.4, and from −26.6 to 15.0 mm on the north, east and up components, respectively. The average value of the mean of the differences is below 1 mm on all three components (see in Table 4), indicating no systematic discrepancies between Wuhan University and GFZ results. The variation of the RMS of differences gets amplified with the increasing length of baselines. The RMS of differences are 6.3, 7.6, and 23.5 mm on the north, east and up components, respectively, indicating no significant differences in view of the accuracy of BDS positioning results, and no systematic discrepancies between results resolved from two types of satellite ephemeris.

### 4.2. Influence of BDS MEO Satellites Distribution on Positioning performance

We analyzed the contribution of MEO satellites distribution on influencing BDS positioning performance due to currently incomplete constellation distribution, which will be fulfilled in 2020. With the same strategy of data processing in Section 2, we reprocessed BDS observations without four MEO satellites. The mean and RMS of baselines differences between BDS positioning results with and without MEO satellites are shown in Figure 8 and Figure 9 in red dots. Comparing to the impact from different ephemeris, the contribution from four MEO satellites is significant on influencing BDS positioning performance. The variation of baseline differences is amplified linearly with the increasing length of baselines, which is insignificant in the ephemeris comparison, thus indicating that the impact on the accuracy of BDS positioning results from ephemeris is stochastic. The RMS of baselines differences also linearly increase with the increasing length of baselines, which shows a similar pattern in the case of different ephemeris, but with larger amplification. The mean and RMS of baselines in differential position time series between BDS results with and without MEO satellites are shown in Table 4. The mean values are less than 1 mm for both north and up components, while it is 5.1 mm on the east component, which is considerable compared to the case of different ephemeris. All baselines are moving eastward without including MEO satellites, especially for baselines longer than 2500 km. The RMS of baselines differential time series is 12.0, 14.1, and 42.9 mm for north, east and up components, respectively. The average value of RMS without MEO satellites is almost two times larger than the case with MEO satellites included, and two times larger than the accuracy of BDS positioning too. The results indicate the utmost importance of MEO satellites in improving the accuracy of BDS positioning, and thus infer that the accuracy of BDS positioning may be improved with increasing the number of MEO satellites.

## 5. Conclusions

Based on BDS/GPS data in CMONOC, we analyzed the positioning accuracy of BDS, GPS and BDS/GPS combined solutions, presented the status of observing and the quality of observation of BDS satellites, depicted the spatial pattern of the position differences between BDS and GPS results in China, discussed the accuracy and its prospect on crustal deformation domain for velocity field derived from BDS data, and deduced the influencing factors on determining the accuracy of BDS positioning. Some conclusions can be drawn as follows.

The SNR and MPD are highly correlated with the elevation angles of satellites, and the higher the elevation angle, the lower the MPD. The MPD for different frequencies are similar with each other, while the SNR are significantly distinct. BDS satellites have similar MPD with GPS satellites. The SNR for IGSO and MEO sets of BDS satellites are similar with GPS satellites, while it is lower than any other satellites for GEO satellites.

The accuracy of BDS positioning results is lower than GPS and BDS/GPS results, with values being 7 and 22 mm on horizontal and vertical components, respectively. The horizontal accuracy for BDS position time series declines with the rising of the latitude in CMONOC, while the vertical one shows spatially random features. The accuracy of velocity field derived from BDS position time series with data volume being about 2.5 years, are 1.2, 1.5, and 4.1 mm/year for north, east and up components. The annual signals with remarkable amplitudes can be emerged and well estimated in BDS position time series. It is insufficient to precisely depict vertical crustal movements in China mainland by 2.5 years of BDS observations. 

The position differences originated from different BDS ephemeris are at the same level with the accuracy of BDS positioning results, indicating that the accuracy of ephemeris is an important factor in determining the accuracy of BDS positioning results. Compared with the results without four MEO satellites, the accuracy of BDS positioning results is doubled with four MEO satellites included in the BDS constellation, thus indicating the other factors in determining the low accuracy of BDS positioning is the small amount of MEO satellites. With the decoding of more MEO satellites and the gradual accomplishing of the MEO constellation, we will look further into the contribution of MEO satellites on the accuracy of BDS positioning results. 

## Figures and Tables

**Table 1:** The average RMS of residuals position time series for BDS, GPS, and GPS/BDS combined solutions, respectively.

Satellite	North (mm)	East (mm)	Up (mm)
BDS	6.1	7.1	22.1
GPS	2.8	2.9	5.8
BDS/GPS	2.4	2.2	5.0

**Table 2:** The mean and RMS of differences between BDS and GPS residual position time series

Title	North (mm)	East (mm)	Up (mm)
mean	−0.8	−1.5	−5.5
RMS	6.8	7.3	21.7

**Table 3:** The average uncertainty of three-dimensional velocity field for BDS and GPS data.

Satellite	North (mm/year)	East (mm/year)	Up (mm/year)
BDS	1.2	1.5	4.1
GPS	0.3	0.3	0.7

**Table 4:** The mean and RMS of baselines differences between BDS positioning results.

Title	North (mm)		East (mm)		Up (mm)	
	Mean	RMS	Mean	RMS	Mean	RMS
Different satellite ephemeris	−0.1	6.3	−0.7	7.6	−0.5	23.5
With and without MEO satellites	0.4	12.0	5.1	14.1	0.0	42.9

## Supplementary Materials

The following are available online at http://www.mdpi.com/1424-8220/18/10/3353/s1, Figure S1: Sky plots (azimuth vs. elevation) for BDS and GPS satellite systems at YNSM on the third day of 2017. (a) BDS; (b) GPS; (c) BDS and GPS; Figure S2: The MPD variations with respect to the elevation angle of satellites. The blue is for the C01 satellite, the green is for the C07 satellite, the red is for the C12 satellite, and the black is for the G11 satellite; Figure S3: The SNR variations with respect to the elevation angle of the satellite. The meanings of the different colors in the diagram are the same as Figure S3; Figure S4: The residual position time series at different stations for BDS, GPS and GPS/BDS combined solutions, respectively. (a) Station AHBB, (b) Station QHGC, (c) Station CHUN, and (d) Station XJBY.
