# Supplementary material for: Positioning Performance of BDS Observation of the Crustal Movement Observation Network of China and Its Potential Application on Crustal Deformation
Source: Sensors (Basel). 2018 Oct 8;18(10):3353. doi: 10.3390/s18103353 (PMC6210552; doi:10.3390/s18103353)
Supplement: Supplementary file 1 [file sensors-18-03353-s001.pdf]

Supplementary Information:

Figure S1. Sky plots (azimuth vs. elevation) for BDS and GPS satellite systems at YNSM on the third day of 2017. (a) BDS; (b) GPS; (c) BDS and GPS.

Figure S2. The MPD variations with respect to the elevation angle of satellites. The blue is for the C01 satellite, the green is for the C07 satellite, the red is for the C12 satellite, and the black is for the G11 satellite.

Figure S3. The SNR variations with respect to the elevation angle of the satellite. The meanings of the different colors in the diagram are the same as Figure s3.

Figure S4. The residual position time series at different stations for BDS, GPS and GPS/BDS combined solutions, respectively. (a) Station AHBB, (b) Station QHGC, (c) Station CHUN, (d) Station XJBY.

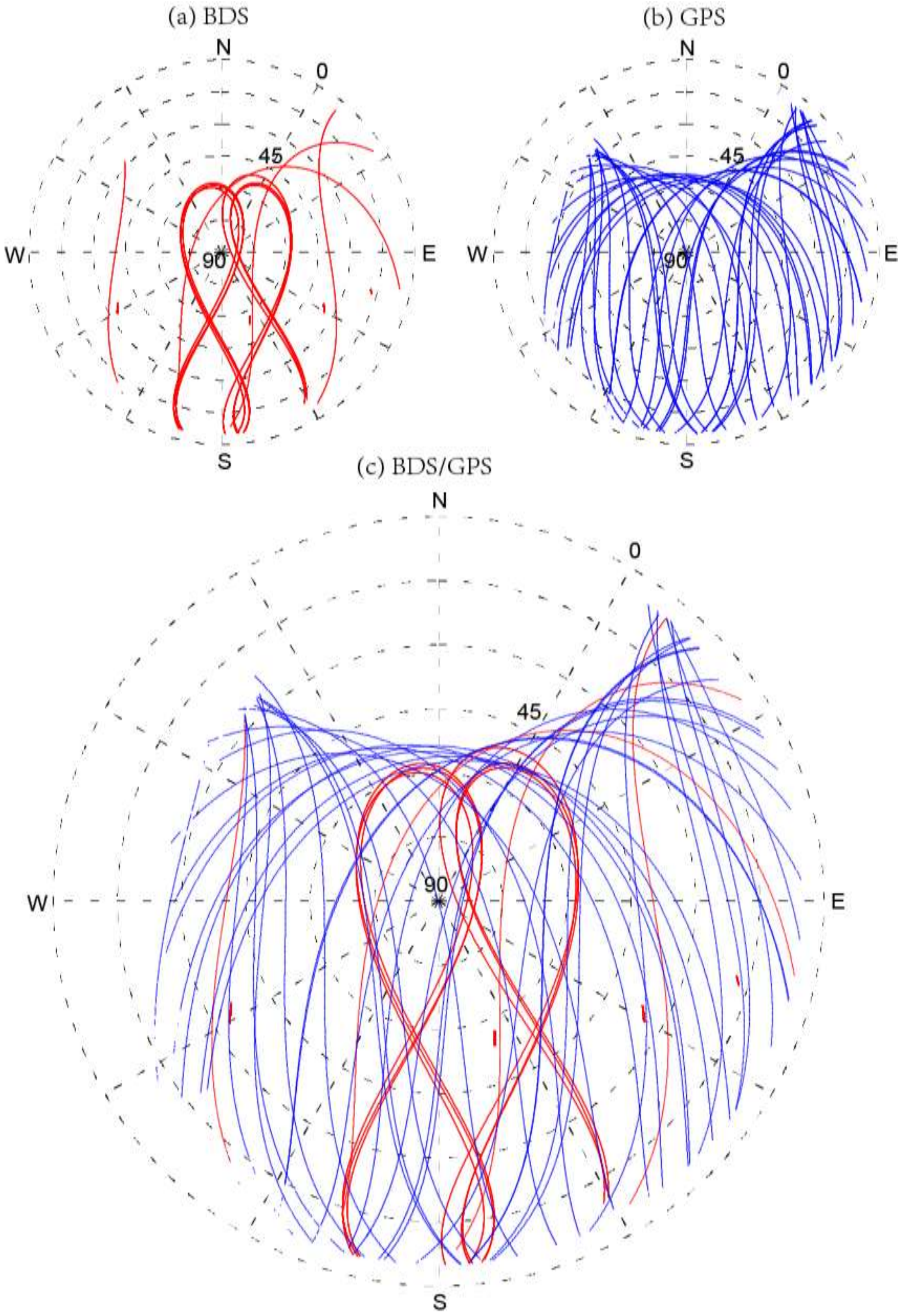

Figure S1. Sky plots (azimuth vs. elevation) for BDS and GPS satellite systems at YNSM on the third day of 2017. (a) BDS, (b) GPS, (c) BDS and GPS.

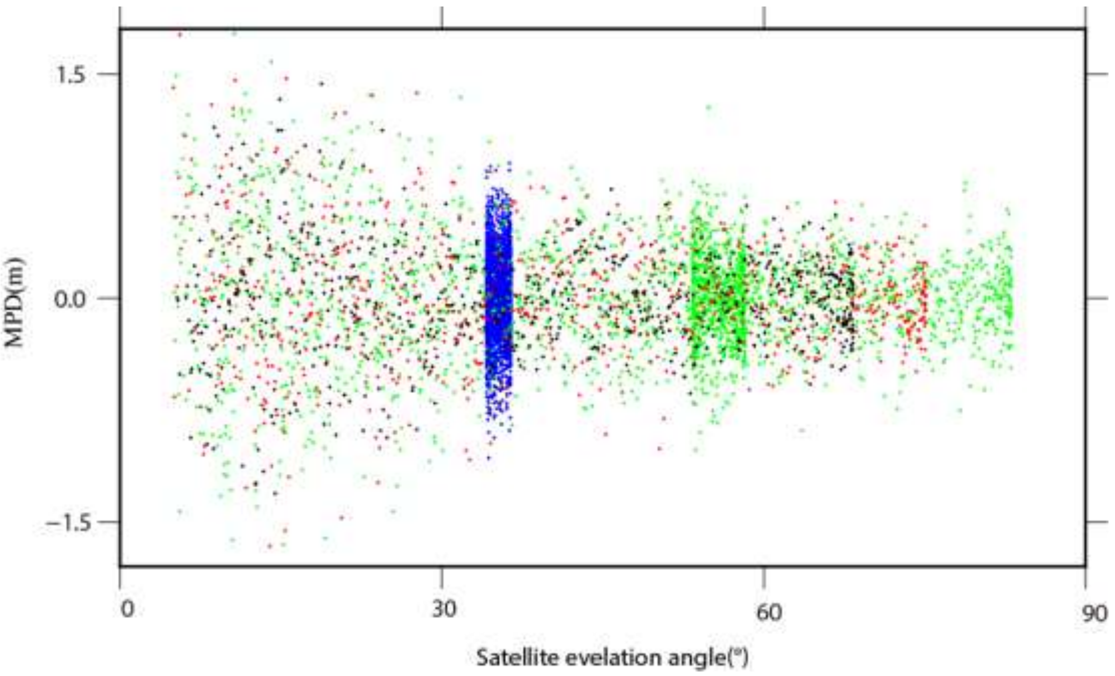

Figure S2. The MPD variations with respect to the elevation angle of satellites. The blue is for the C01 satellite, the green is for the C07 satellite, the red is for the C12 satellite, and the black is for the G11 satellite.

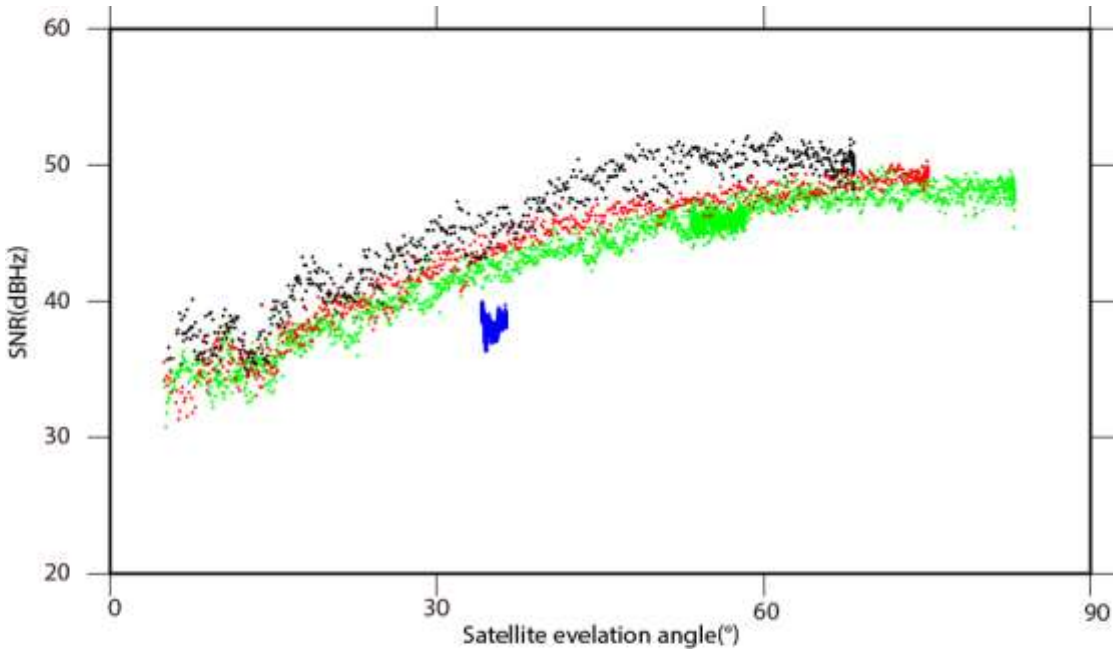

Figure S3. The SNR variations with respect to the elevation angle of the satellite. The meanings of the different colors in the diagram are the same as Figure s3.

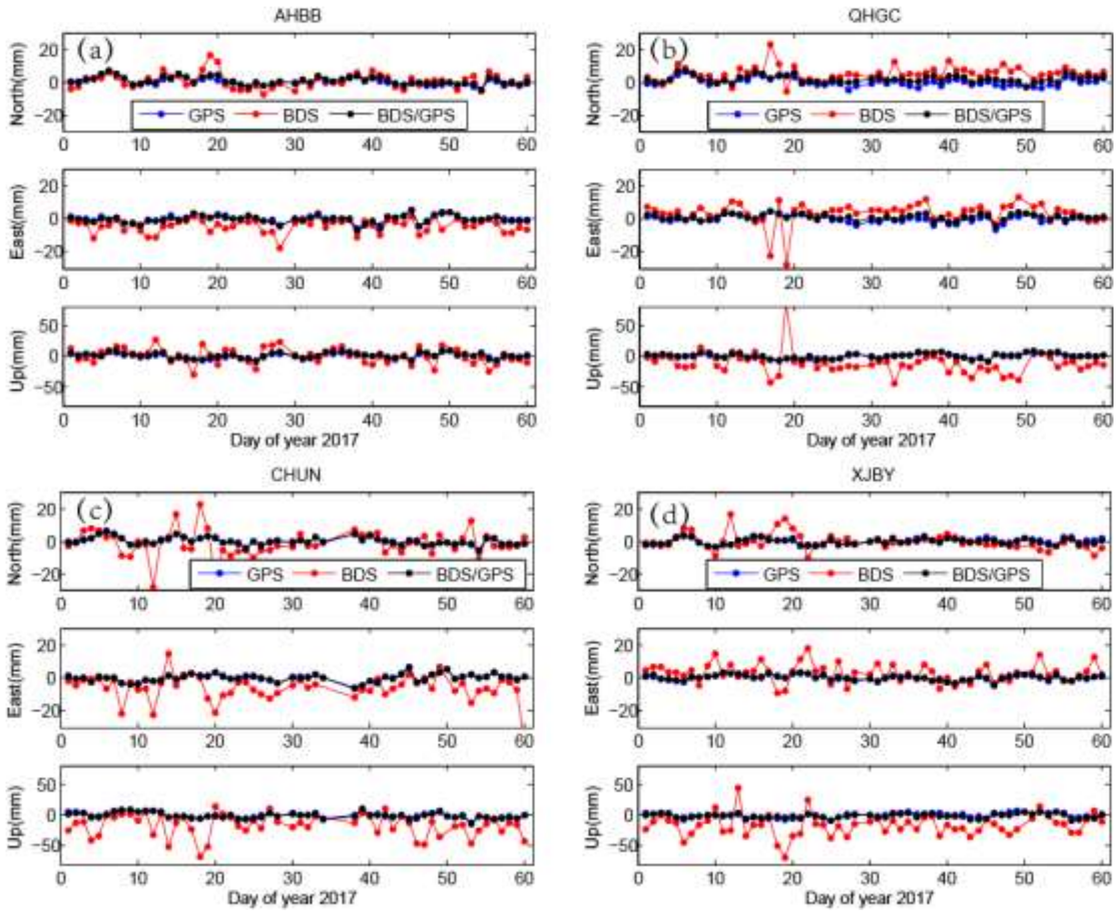

Figure S4. The residual position time series at different stations for BDS, GPS and GPS/BDS combined solutions, respectively. (a) Station AHBB, (b) Station QHGC, (c) Station CHUN, (d) Station XJBY.
